# Use of Winemaking By-Products for the Functionalization of Polylactic Acid for Biomedical Applications

**DOI:** 10.3390/antiox12071416

**Published:** 2023-07-13

**Authors:** Lidia Verano-Naranjo, Cristina Cejudo-Bastante, Lourdes Casas, Enrique Martínez de la Ossa, Casimiro Mantell

**Affiliations:** Chemical Engineering and Food Technology Department, Science Faculty, Wine and Agrifood Research Institute (IVAGRO), University of Cadiz, Puerto Real, 11510 Cadiz, Spain; lidia.verano@uca.es (L.V.-N.); cristina.cejudo@gm.uca.es (C.C.-B.); lourdes.casas@uca.es (L.C.); enrique.martinezdelaossa@uca.es (E.M.d.l.O.)

**Keywords:** grape pomace, supercritical impregnation, soaking, polylactic acid, polyphenols, antioxidants

## Abstract

The addition of naturally active compounds to implantable polymers is an efficient strategy against inflammation issues that might lead to rejection, while promoting controlled re-endothelialization of the tissues. This work proposes the use of winemaking by-products with high active properties of biomedical interest to obtain bioactive PLA by using supercritical technologies. First, two red grape pomace extracts, obtained by high-pressure extraction with supercritical CO_2_ and cosolvents (either ethanol or water–ethanol), have been studied. Second, two impregnation methods have been studied with both extracts, traditional supercritical CO_2_-assisted impregnation (SSI) and a novel pressurized soaking method (PSI). The amount of extract impregnated as well as the bioactivity levels achieved—i.e., antioxidant, antimicrobial, and anti-inflammatory properties— have been determined for each extract and impregnation method at different pressure and temperature conditions. Both extracts obtained had good antioxidant, anti-inflammatory, and antibacterial capacities, especially the hydroethanolic one (0.50 ± 0.03 mg TE/g versus 0.24 ± 0.03 mg TE/g, respectively). Regarding impregnated filaments, impregnation loadings depended especially on the extract and P/T conditions, providing up to 8% (extract mass/polymer mass) of impregnation. The antioxidant capacity increased noteworthily by using the ethanolic extract by PSI, with values near 100 µg TE/g PLA.

## 1. Introduction

Grape pomace is mostly formed by a mixture of seeds and peels from *Vitis vinifera* fruits that account for between 15 and 20% of the total by-products of the wine industry and whose world production may reach close to 1200 tons per year [1,2]. It is rich in polyphenols, mostly phenolic acids, flavonoids (anthocyanins, catechins, and flavonols), and procyanidins. Hence, grape pomace extracts (GPEs) exhibit nutraceutical, antimicrobial, antioxidant, anti-inflammatory, and other interesting properties that make them suitable to obtain products with a high added value for the food, pharmaceutical, and biomedical industries, while contributing to a circular economy. In the biomedical field, in particular, GPEs have demonstrated their anti-platelet and cardioprotective effects [3,4,5]. Sánchez-Gomar and coworkers [6] found that ethanolic and aqueous GPEs had pro-angiogenic and anti-apoptotic properties over endothelial colony-forming cells. Furthermore, the anti-proliferative and pro-apoptotic properties of GPEs on certain cancerous cells were noticed by Caponio and coworkers [7]. These characteristics could make GPEs attractive substances to be added to prolonged-release implants such as coronary stents. The progressive release of these active substances could promote endothelization while avoiding excessive cell growth that might lead to rejection or restenosis.

Drug-release devices have been traditionally produced either by adding the active compounds (API) during the polymerization steps or by soaking the final polymeric device in a solution that contains the active compounds [8]. Choosing the right solvent is one of the key aspects of the soaking method, because it must have the capacity, on the one hand, to solubilize the substance of interest and, on the other, to cause the swelling of the polymer so that the substance can diffuse and be retained inside the polymer structure due to chemical interactions [9,10]. In fact, normally this technique employs organic solvents, sometimes in large amounts, to increase the soaking capacity. Hence, novel and more sustainable technologies have been developed in recent years to avoid or minimize the use of organic solvents. In relation to this, supercritical CO_2_ (scCO_2_) methodologies have been increasingly applied to the loading of drug-active compounds into polymeric matrices intended to be used as active medical devices or delivery systems [11]. In supercritical solvent impregnation (SSI), the ability of CO_2_ in the supercritical state (above 31,8 v and 73 bar) to penetrate through porous matrices, such as those of polymers, is leveraged to impregnate the desired active substances that can be effectively solubilized and carried by this supercritical solvent. At the end of the process, the system pressure drops and the CO_2_ returns to a gaseous state, while the active ingredients remain trapped inside the matrix. The main advantage of this technique is that it is not necessary to use organic solvents as long as the compounds of interest are highly soluble in scCO_2_. Nevertheless, in the case of less soluble compounds, small amounts of a polar solvent, such as ethanol, may be used to enhance the process [12]. Given that the polymer is not initially in contact with the API solution, the supercritical CO_2_ must effectively solubilize and transport the active compounds into the polymeric device when this method is used. Therefore, the impregnation efficiency is mainly dependent on the good solubility of the compounds in the supercritical phase.

In this work, a novel impregnation method is proposed, the pressurized soaking impregnation (PSI) method. This method combines the SSI and the traditional soaking method. The polymer is directly submerged into the active solution and CO_2_ is applied at a high pressure. This enhances the impregnation efficiency thanks to the combined action of the solvent–polymer direct contact and the effect of the high pressure. This effect results in the swelling of the polymer and a better impregnation of the compounds that exhibit a higher polarity and were less impregnated by SSI.

Two different grape pomace extracts were produced (an ethanolic extract and a hydroethanolic extract) to be impregnated into polylactic acid polymer filaments through two high-pressure techniques, namely SSI and PSI. Even though other research studies have already functionalized this polymer type by SSI using either drugs [9,13] or other natural ingredients, such as mango leaf [14] or olive leaf extract [15], no investigations have been conducted on the use of grape pomace extracts to produce polymeric devices intended for biomedical applications. Therefore, the objective of the present study is to determine how two different extracts, two impregnation methods, and variations in the operating pressure and temperature may have a relevant influence on the impregnation loadings and on the bioactivity capacity of the final polymeric devices.

## 2. Materials and Methods

### 2.1. Raw Materials and Chemicals

The pomace from the pressing of the Tempranillo grape variety to produce rosé wine was used as the raw material to obtain the extracts. The grapes had been cultivated in the south of Spain and were harvested and processed in September 2021. After the pressing of the grapes, the pomace was frozen for storage. Later on, it was dried and grilled before its utilization.

The polylactic acid filament used for the impregnations was supplied by Mundo Reader S.L. (Madrid, Spain). This polymer is a colorless filament of 100% PLA with a 1.24 g/cm^3^ density, a nominal diameter of 1.75 mm, a fusion temperature of 145–160 °C, and a glass transition temperature of 56–64 °C (according to the specifications provided by the manufacturer).

Other reagents and microbial strains used are specified in Table 1.

### 2.2. Producing the Extracts

Two different grape pomace extracts were used for this study: an ethanolic GPE and a hydroethanolic GPE. Except for the solvent used, both extracts were obtained using the same method. The phenolic content in the extract depends on the nature of the solvents used. Ethanol has already been demonstrated to be a good solvent for the recovery of active compounds from winemaking by-products. Nevertheless, an ethanol–water mixture has also been demonstrated to improve the extraction of anthocyanins, flavonols, and flavanols [16]. Therefore, both solvents were employed in order to determine any possible differences with regard to the subsequent impregnation process into the polymeric matrix. The extracts were obtained by means of a supercritical extraction instrument supplied by Thar Technologies (Pittsburgh, PA, USA) equipped with a 500 mL extraction vessel fitted with an electric heating jacket, a high-pressure pump, and a back pressure regulator to maintain the set conditions. The system was operated in batch mode and, for each extraction, approximately 70 g of grape pomace was placed inside the extraction vessel together with 400 mL of solvent (ethanol in the case of the ethanolic GPE and an equivolumetric mixture of water/ethanol for the hydroethanolic GPE). Then, the vessel was heated, and CO_2_ was pumped in until the desired operating conditions—55 °C and 200 bar—were reached. These operating conditions were selected based on the optimal values determined by earlier studies to obtain the greatest extraction yields and the highest antioxidant capacity of the red grape pomace extracts [17]. After 1 h in static mode, the system was depressurized and cooled down. The resulting extracts were stored at 4 °C until their use.

### 2.3. Phenolic Characterization of Extracts

The identification and the quantification of polyphenols in the grape pomace extracts were carried out by Ultra-High-Performance Liquid Chromatography–Electrospray Ionization–Time of Flight–Mass Spectrometry (UHPLC-ESI-ToF-MS) using a Xevo G2-S system supplied by Waters Corporation (Milford, MA, USA). For all the analyses, a UPLC BEH-C18 column (2.1 × 100 mm, 1.7 µm) and a 5 µL injection volume were employed. Flavanols and flavonols were detected and determined using a binary solvent system (A: 0.1% formic acid in water; B: 0.1% formic acid in acetonitrile) pumped at 0.5 mL/min. A column oven temperature of 45 °C was used. The volumetric composition gradient of the mobile phase was established as follows: 97% of A at the initial time, 90% of A at 3.5 min, 85% of A at 5.0 min, 80% of A at 6.5 min, 75% of A at 8.0 min, 60% of A at 9.5 min, 50% of A at 10.5 min, 25% of A at 11.5 min, 0% of A from 12.5 to 13.5 min, and 97% of A at 14.0 min. The electrospray was operated in negative ionization mode for a full scan analysis (100–1200 Da), with a cone voltage of 40 V, a capillary voltage of 0.7 kV, a source temperature of 120 °C, and a desolvation temperature of 850 °C. For anthocyanin detection, the same UHPLC column and injection volume were used at a solvent flow of 0.4 mL/min. A was composed of 0.1% formic acid in water and phase B was 100% methanol. The following solvent gradient was applied: 95% of A at the initial time, 85% of A at 3.0 min, 75% of A at 4.8 min, 60% of A at 6.0 min, 55% of A at 7.0 min, 0% of A from 9.0 to 12.0 min, and 95% of A at 15.0 min. The electrospray was operated in positive ionization mode for a full scan analysis (100–1200 Da), while the rest of the parameters remained unchanged.

The phenolic compounds were identified based on their mass spectra and retention times according to the bibliography. They were quantified according to the calibration line of each target compound phenolic family, at concentrations from 1 to 100 µg/mL (Table 2).

### 2.4. Determination of Extracts’ Bioactivity

In order to determine the bioactivity of both extracts, a number of spectrophotometric tests were carried out using a Synergy HTX Multi-Mode Microplate Reader by BioTek Instruments (Winooski, VT, USA).

#### 2.4.1. Antioxidant Capacity

The antioxidant capacity of both extracts was determined through 2,2-diphenyl-1-picrylhydrazyl (DPPH) and 2,2′-azinobis-(3-etilbenzotiazolina-6-sulfonic) acid (ABTS), as both of these methods are widely used to measure the scavenging capacity of antioxidants. The DPPH assay is based on the one proposed by Brand and Williams [18] with modifications. In this case, 7 µL of the extracts at different concentrations (ranging from 5 to 225 µg/mL) was mixed with 293 µL of 6·10^−5^ M DPPH ethanolic solution. The absorbance at 515 nm was measured after 150 min in the absence of light, i.e., when the reaction reaches a stationary state.

For the ABTS procedure, based on the one proposed by Re and coworkers [19], an aqueous solution was prepared by mixing ABTS reagent and K_2_S_2_O_8_ at final concentrations of 7 and 2.45 mM of each one, respectively. This solution was then left to stand in the absence of light for at least 16 h to allow the formation of the ABTS free radicals. Then, this aqueous solution was diluted in ethanol until an absorbance of approximately 0.7 at 750 nm (the maximum wavelength registered for this radical in ethanolic solution) was obtained. For the analyses, 10 µL of the extracts at different concentrations (ranging from 5 to 225 µg/mL) was mixed with 1 mL of an ethanolic ABTS solution and the absorbance at 750 nm was measured after 30 min, when the reaction had reached a stable state.

In both methods, oxidative inhibition (OI) is related to the difference in absorbances according to Equation (1), where ${Abs}_{t}$ is the absorbance of each test sample at the corresponding wavelengths and ${Abs}_{o}$ is the absorbance of a control sample where the extract has been replaced by ethanol. The final concentration of the samples was plotted versus the OI and the concentration of extract necessary to inhibit 50% of oxidation (IC_50_) was estimated. Both measurements were also carried out using Trolox (6-hydroxy-2,5,7,8-tetramethylchroman-2-carboxylic acid) as a standard, so that the results could be expressed as Trolox-equivalent mass.

(1) $$OI\left(\%\right)=\frac{{Abs}_{o}-{Abs}_{t}}{{Abs}_{o}}\times 100$$

#### 2.4.2. Anti-Inflammatory Capacity

The anti-inflammatory capacity of the extracts was determined by two spectrophotometric measurements as follows: the power to inhibit the thermal denaturalization of proteins and the ability to inhibit the lipoxygenase activity. First of all, the GPE extracts were dried and re-diluted in water, because an ethanolic base could interfere with the measurements.

Inflammatory processes have an influence on the denaturation of proteins and the inhibition of this phenomenon is one of the main mechanisms of action of non-steroidal anti-inflammatory drugs [20]. The ability of natural extracts to prevent the thermal and hypotonic denaturation of proteins may explain their anti-inflammatory properties. It has been suggested that the extract could have the capacity to inhibit the release of neutrophil lysosomal components, which are enzymes and proteinases which, when they are extracellularly released, can cause further inflammation and tissue damage [21,22]. In order to determine the capability of the GPEs to prevent the denaturation of egg albumin, 0.2 mL of egg albumin solution (prepared mixing 0.5 g of powered egg albumin and 10 mL of distilled water), 2.8 mL of phosphate buffer saline (PBS) pH 6.3, and 2 mL of every extract at different concentrations (60–1800 µg/mL) were incubated at 37 °C for 15 min and then at 70 °C for 5 min. After cooling, the absorbance of the supernatant was measured at 660 nm. The same reaction using distilled water instead of the extract was used as the control. The background absorbance of the extracts was also measured by mixing 3 mL of PBS with 2 mL of each extract dilution. The denaturation inhibition (DI) was calculated through Equation (2), where ${Abs}_{o}$ is the absorbance of the control, ${Abs}_{t}$ is the absorbance of each test sample and ${Abs}_{b}$ is the background absorbance of the corresponding test sample. IC_50_ is the concentration of extract necessary to inhibit the protein denaturation by 50%.

(2) $$DI\;\left(\%\right)=\left(1-\frac{{Abs}_{o}}{{Abs}_{t}-{Abs}_{b}}\right)\times 100$$

Lipoxidases or lipoxygenases (LOXs) are key enzymes for the biosynthesis of leukotriene. Arachidonic acid is cleaved from membrane phospholipids and can be converted to leukotrienes and prostaglandins via the lipoxygenase and cyclooxygenase pathways, respectively. That is, LOXs are responsible for catalyzing the deoxygenation of polyunsaturated fatty acids to produce hydroperoxides with a conjugated diene structure, such as that of leukotrienes [23]. Polyphenols can interfere with the arachidonic acid metabolism by inhibiting lipoxygenase activity and they can scavenge the free radicals produced during the process. The LOX inhibitory action was estimated by means of FOX reagent (ferrous oxidation-xylenol orange) and a colorimetric determination. In the presence of hydroperoxides, Fe^2+^ is oxidized to Fe^3+^, which in turn oxidizes orange xylenol into a bluish-colored complex [24]. For this procedure, 20 µL of extract samples at different concentrations (75–3000 µg/mL), 20µL of 50 mM Tris-HCl buffer pH 7.4, and 40 µL of lipoxidase (10,000–20,000 units/mL) prepared in the same buffer were preincubated at room temperature for 5 min. Then, 40 µL of 140 µM buffered linoleic acid was added and incubated in the absence of light at room temperature for 25 min. After that, 100 µL of FOX reagent (30 mM sulfuric acid, 100 µM xylenol orange, 100 µM ferrous sulphate in methanol/water 9:1) was added and, after 30 min in the dark at room temperature, absorbance was measured at 560 nm. The control sample was tested by replacing the extract with distilled water. In all cases, a blank test was carried out in order to determine any possible activity in the absence of a substrate during the incubation period. The lipoxygenase inhibition (LI) was calculated following Equation (3), where ${Abs}_{t}$ is the absorbance of the test sample, ${Abs}_{b}$ is the absorbance of the blank of the test sample, ${Abs}_{o}$ is the absorbance of the control, and ${{Abs}_{o}}_{b}$ is the absorbance of the blank of the control. In this case, IC_50_ is the concentration of the extract required to inhibit the hydroperoxides’ formation by 50%.

(3) $$LI\;\left(\%\right)=\left(1-\frac{{Abs}_{t}-{Abs}_{b}}{{Abs}_{o}-{{Abs}_{o}}_{b}}\right)\times 100$$

#### 2.4.3. Antimicrobial Capacity

The antimicrobial capacity of the two GPEs against *S. aureus* was determined through a microtiter plate assay using the chromogenic marker 2,3,5,-triphenyltetrazoliumchloride (TTC). For the test, 200 μL of 10^6^ CFU/mL adjusted inoculum of the bacteria grown in Tryptic Soy Broth (TSB) medium and 20 μL of every extract at different concentrations (5–100 μg/mL) were incubated for 24 h at 36 °C. Then, 10 μL of 5 mg/mL TTC was added and absorbance at 500 nm was measured after 30 min at 36 °C. During this second incubation time, TTC is reduced in the presence of bacteria into red formazan, which indicates the activity and viability of the cells [25]. Furthermore, a positive control (200 μL bacteria 10^6^ CFU/mL in TSB medium + 20 μL ethanol + 10 μL TTC) and a blank of the ethanolic dilutions of the extracts (200 μL bacteria 10^6^ CFU/mL in TSB medium + 20 μL ethanol + 10 μL TTC) were carried out in the same way. The percentage of inhibition of the bacteria growth, antimicrobial inhibition (AI), was calculated by Equation (4), where ${Abs}_{t}$ is the absorbance of the test sample, ${Abs}_{b}$ is the absorbance of the blank of the test sample, ${Abs}_{o}$ is the absorbance of the positive control, and ${{Abs}_{o}}_{b}$ is the absorbance of the blank of the control. The minimum inhibitory concentrations (MICs) required to inhibit the bacteria growth by 90% with respect to that of the positive control test were determined.

(4) $$AI\;\left(\%\right)=\left(1-\frac{{Abs}_{t}-{Abs}_{b}}{{Abs}_{o}-{{Abs}_{o}}_{b}}\right)\times 100$$

### 2.5. Polylactic Acid Impregnation

Two different methods were used to impregnate the polymer with each one of the two extracts previously obtained. For all the impregnations, a supercritical impregnation instrument provided by Waters Corporation (Milford, MA, USA) equipped with a 100 mL electric-jacketed impregnation cell with a high-pressure pump and a back pressure regulator to maintain the set conditions was used.

For the first series of experiments, a regular supercritical solvent impregnation (SSI) was conducted. A 3 mL volume of the extract was placed at the bottom of the impregnation cell. Then, nine sections of PLA filament (all of them 30 mm long, 0.08 g mass, and an average diameter of 1.68 mm) were placed in a steel basket support inside the vessel. The purpose of the steel support was to prevent any direct contact between the polymer and the extract. The cell was then closed and heated, and CO_2_ was pumped at 10 g/min until the desired conditions were reached. The system was maintained under these conditions for two hours and afterward it was depressurized at a rate of 40 bar/min.

For the second round of experiments, the polymer was immersed into the extract for the pressurized soaking impregnation (PSI). Nine sections of PLA filament (with the same characteristics as the ones used for the previous experiments) were placed at the bottom of the impregnation cell together with 10 mL of extract. The amount of extract had to be increased to make sure that all the surfaces of the polymer portions were in direct contact with the extract. The experimental procedure was the same as for the previous series of experiments.

The effect of the operating temperature and pressure conditions in the impregnation of ethanolic and hydroethanolic extracts into PLA following the two different described methods was determined. A multifactorial level design of experiments was followed according to Table 3. Three pressure levels were considered (100, 250, and 400 bar) from the whole range of CO_2_ supercritical conditions depending on the operational capacity of the equipment, along with two temperature levels (35 and 55 °C) as, under certain conditions, PLA melts when exposed to higher temperatures and the GPEs could also become thermally degraded.

### 2.6. Impregnated Samples’ Characterization

When a solvent is in contact with a polymer, it may penetrate the empty space between the polymeric chain network, which may in turn produce a movement of these chains and swell the polymer. The structural modification that takes place in the polymeric filaments after being in contact with the solvent and the carbon dioxide under supercritical conditions has been evaluated, and the permanent swelling or expansion of the polymer after the treatment has been quantified. For this purpose, the filament volume was measured before ($V_{i}$) and some days after the impregnation process had been completed ($V_{f}$) in order to let any traces of the remaining CO_2_ diffuse, as well as to allow the permanent swelling to stabilize [26]. The percentage of expansion of the polymer was calculated by Equation (5).

(5) $$Expansion\left(\%\right)=\frac{V_{f}-V_{i}}{V_{i}}\cdot 100$$

Additionally, some of the samples were subjected to Scanning Electron Microscopy (SEM) by means of a Nova NanoSEM 450 microscope supplied by FEI (Hillsboro, OR, USA). Previous to their examination, the samples were coated with a thin layer of gold (about 15 nm) using a Cressington Sputter Coater model 208 HR from Cressington Scientific Instrument (Watford, UK) to improve their conductivity for better imaging. The samples were observed under vacuum, with a high voltage of 5.00 kV, and with an Everhart-Thornley detector in secondary electron mode. The magnification level used was 2000×.

The amount of the GPEs impregnated in the PLA samples was determined by a gravimetry. For this purpose, the mass of each sample was measured before ($m_{i}$) and after its impregnation ($m_{f}$) (see Equation (6)). Likewise, their final masses were measured again several days after the process had been completed in order to allow enough time for the carbon dioxide trapped inside the polymer to be released.

(6) $$GPE\;loading\;\left(\%\right)=\frac{m_{f}-m_{i}}{m_{i}}\cdot 100$$

The bioactivity of the generated samples was evaluated through their antioxidant activity. A 30 mg portion of impregnated PLA was submerged into 300 µL of the 6×10^−5^ M ethanolic DPPH solution and allowed to react for 150 min in the absence of light at room temperature. The equivalent Trolox amount released during the time of each reaction was determined by measuring, at the appropriate wavelength, their absorbance difference with respect to the unimpregnated polymer in the same way as for the extracts.

### 2.7. Statistical Analysis

All analytical determinations were performed at least in triplicate. The results were denoted as means and standard deviations.

The multifactorial level design of experiments indicated in Table 3 was applied to study the variable influences over the expansion, the GPE loading, and the antioxidant capacity of the impregnated devices. The ANOVA multifactorial study (*p* < 0.05) was performed by means of Statgraphics 19® software (Statgraphics Technologies Inc., The Plains, VA, USA) and Pareto charts were used to represent the statistical relevance of factors.

## 3. Results and Discussion

### 3.1. Extracts’ Characterization

**Figure 1:**
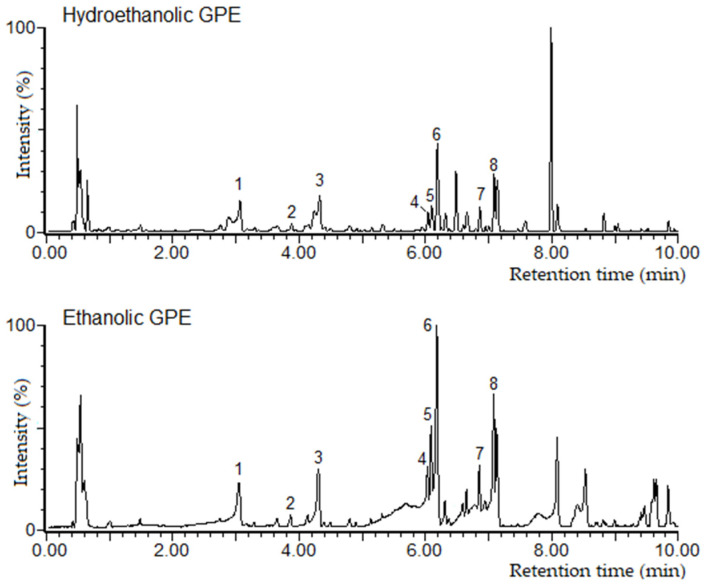
Chromatograms obtained by UPLC-ESI-ToF-MS of the hydroethanolic and ethanolic grape pomace extracts. A 100% intensity corresponds to the highest peak. Identified peaks: 1. Catechin, 2. Procyanidin B, 3. Epicatechin, 4. Quercetin-3-*O*-galactoside, 5. Quercetin-3-*O*-glucuronide, 6. Quercetin-3-*O*-glucoside, 7. Kaempherol-3-*O*-glucoside, 8. Isorhamnetin-3-*O*-glucoside.

**Figure 2:**
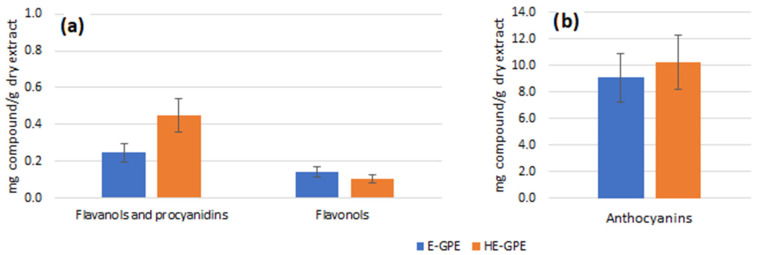
Composition of the ethanolic (E-GPE) and hydroethanolic (HE-GPE) grape pomace extracts per group of polyphenols (*n* = 3): (**a**) Flavanols, procyanidins, flavonols, and (**b**) anthocyanins. Obtained by the sum of individual compounds (Table 4 and Table 5) and related to the extract concentration.

The extracting solvents provided high total extraction yields (33% for ethanolic GPE and 40% for hydroethanolic GPE, measured as mass of dry extract with respect to mass of raw material). The ethanolic GPE showed a concentration of just 81.6 ± 2.9 g/L, while the concentration of the hydroalcoholic extract was much higher, 207.7 ± 5.5 g/L. Figure 1 shows the chromatograms corresponding to each extract and the main compounds detected. Regarding the quantification, Table 4 shows a higher concentration of both flavanols and flavonols detected in the hydroalcoholic extract with respect to the ethanolic one. Although an elevated concentration of anthocyanins was quantified in both extracts, it was higher in the case of the hydroethanolic one (Table 5). The CO_2_ in combination with water or ethanol produces an in situ equilibrium of the formation of carbonic acid and/or alkyl carbonic acid [27] and the decrease in pH improves the diffusivity of anthocyanins [28,29]. The final measured pH of each extract (4.6 for hydroethanolic GPE and 5.1 for ethanolic GPE) could have a positive impact on anthocyanins’ stability, with improved values in the hydroethanolic extract. The anthocyanins found were mainly malvidin derivatives, as they are characteristic of Tempranillo grapes [30,31]. With regard to the proportions of these three families of compounds per dry extract (Figure 2), it can be seen that the hydroalcoholic extract is richer in flavanols, while the percentages of flavonols and anthocyanidins are similar in both extracts. Aqueous mixtures of solvents have been previously proven to be more effective for the extraction of condensed tannins (proanthocyanidins) and anthocyanins than pure solvents [32,33].

Table 6 shows the bioactivity of the two extracts considered. First, their antioxidant capacity according to the DPPH and ABTS methods is expressed as the Trolox equivalent (TE) and the IC_50_ value. When the two extracts are compared, it can be noticed that the hydroethanolic GPE has a higher antioxidant power (a greater quantity of TE and a lower IC_50_). According to several studies where the antioxidant activity of individual polyphenols was compared [34,35], catechins and procyanidins are probably the main ones responsible for the antioxidant capacity of GPEs and these compounds were found in higher proportions in the hydroethanolic extract. By comparing both methods, it can be seen that the antioxidant capacity as determined by ABTS assay was considerably greater than that determined by the DPPH assay, for both extracts. Several authors have found significant discrepancies when applying these two methods to the same compound or mixture. These differences are possibly due to the fact that both radicals react differently with the same compound [36]. Floegel and coworkers [37] proposed that highly pigmented and hydrophilic antioxidants are best revealed by the ABTS assay, while hydrophobic mixtures are best analyzed by the DPPH assay.

Regarding protein denaturation inhibition of the two grape pomace extracts, the tests revealed a poor but incipient anti-denaturing capacity, because the same test on anti-inflammatory drugs like diclofenac revealed an IC_50_ of 625 µg/mL [38]. The hydroethanolic GPE presents an IC_50_ of less than half that of the ethanolic GPE, which means that the former has a much higher inhibition capacity of egg albumin denaturation. Nevertheless, other plant extracts have revealed an anti-denaturant capacity higher than anti-inflammatory drugs. For instance, Osey Akoto and coworkers [39] reported that *Ocimum basilicum Linn*. fruit extracts obtained using hexane and ethanol showed a higher inhibition of protein denaturation than acetylsalicylic acid at concentrations from 1000 to 5000 µg/mL. Conversely, ethanolic GPE presents higher anti-lipoxygenase activity (lower IC_50_) than hydroethanolic GPE. This could be because the lipoxygenase activity was associated with non-polar compounds [40], such as flavons and flavonols, which are present in greater quantities in the ethanolic extract.

Several studies have demonstrated the antimicrobial activity of grape by-products [17,41,42]. In this work, the antimicrobial capacity of each of the two GPEs against *S. aureus*, the principal microorganism responsible for implant infections [43], has been studied. Both grape pomace extracts exhibit similar minimum inhibitory concentrations, 17.0 ± 1.7 and 18.2 ± 0.7 µg/mL, for ethanolic and hydroethanolic GPEs, respectively. Some other authors found similar activities against *S. aureus* for other grape pomace extracts. For example, Silva and coworkers [41] proved a MIC value in the range of 7 and 12 µg/mL for Merlot and Syrah pomace extract. However, Poveda and coworkers [44] obtained Tempranillo pomace extracts by ultrasound-assisted extraction and accelerated solvent extraction with worse activity against this bacterium, an IC_90_ of 2720 and 6470 µg/mL, respectively. According to Sanhueza and coworkers [45], the antibiotic activity of the extracts obtained in the present work is comparable to Chloranphenicol, which presented a MIC of 16 µg/mL against the same microorganism strain.

### 3.2. Evaluation of the Impregnation Process

The expansion of the polymer was the first parameter in the impregnation process that had to be evaluated. It should be noted that the amount of solvent that can be solved into a polymer depends on multiple factors such as the chemical nature of both the polymer and the solvent as well as on a number of experimental conditions, such as temperature, pressure, or pH. This is, therefore, an important parameter that may determine the final application of the product. For example, for the device to be used as a scaffolding, a great expansion or foaming is required, i.e., polymers with a high porosity rate that allow their correct adherence to the tissues. On the other hand, in the case of vascular implants or stents, a high degree of porosity might compromise hemocompatibility [46]. Polylactic acid is a versatile polymer, which has been processed by supercritical fluid to produce either of the two types, foamed structures [47] and low-porosity devices [48].

**Figure 3:**
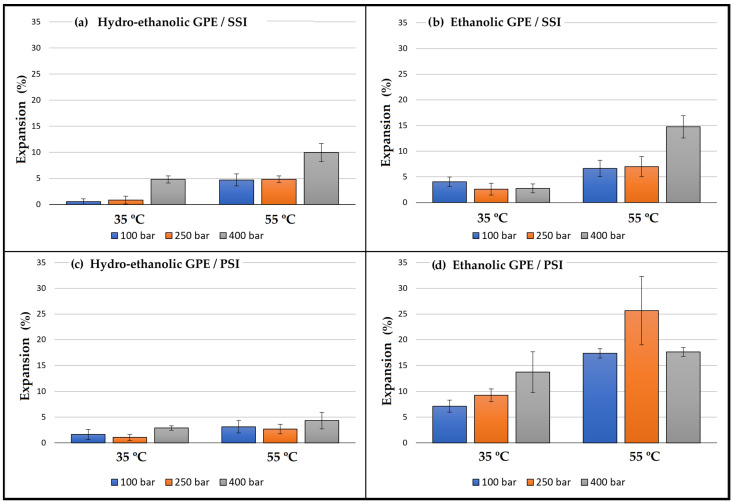
Expansion of the impregnated samples depending on the type of GPE, the impregnation method, and the temperature and pressure conditions. Results are represented by the mean of three values and standard deviation.

**Figure 4:**
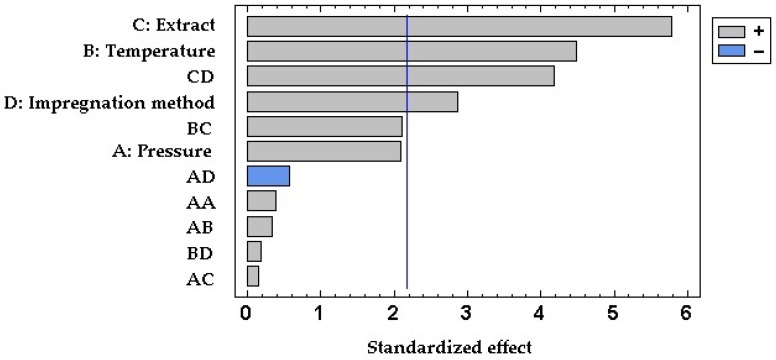
Standardized Pareto chart of the permanent swelling or expansion achieved by the impregnated samples. Effects: pressure (A), temperature (B), type of extract (C), impregnation method (D), and two-factor interactions.

**Figure 5:**
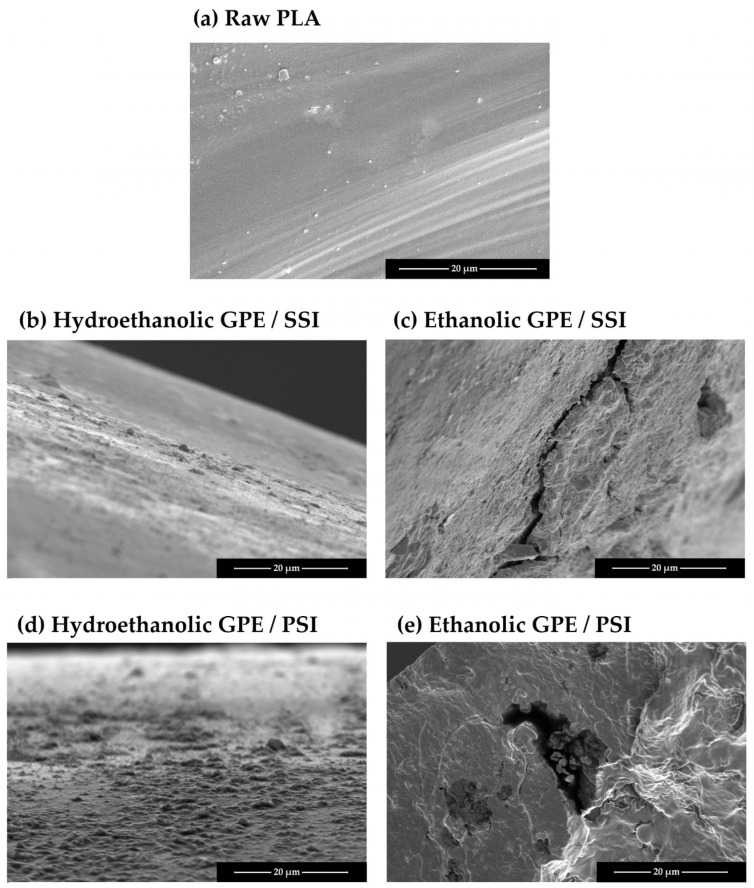
SEM images (×2000) of PLA filaments impregnated with either ethanolic or hydroethanolic grape pomace extracts at 35 °C and 100 bar and using either impregnation method, SSI and PSI.

Figure 3 displays the percentage of expansion of the samples impregnated with both GPEs using the two impregnation methods. As can be observed, the expansion was higher in the samples that were impregnated with the ethanolic GPE in comparison with those impregnated with the hydroethanolic extract. This translates into a significant and positive effect of factor C as can be seen in the Pareto chart in Figure 4. Organic solvents like ethanol can promote PLA plasticizing so that the polymeric chains increase their mobility, and a greater swelling may occur [14]. In view of the SEM images displayed in Figure 5, it appears that the surface of the polymer breaks when impregnated using the ethanolic extract. This produces a number of channels and pores that enhance the swelling effect of the polymer in comparison to that achieved by hydroethanolic impregnation, which seems to have a lesser effect.

Concerning the impregnation method, the contact of the polymer samples with the extract in the PSI incremented the expansion in the case of ethanolic GPE. This may be due to a greater sorption of ethanol by the polymer when in direct contact, which improves its plasticizer effect. On the other hand, when impregnation occurred in contact with hydroethanolic GPE, the permanent swelling was similar or even lower than when using SSI impregnation. In this case, because PLA is in contact with a hydroalcoholic solution, the polymer may start a hydrolytic degradation process. This hydrolysis depends on the presence of water molecules in the polymer matrix, as the presence of ethanol and its swelling effect may favor the sorption of the water [49], which may also be enhanced by the high pressures applied. The shortening of the polymeric chains may reduce the free volume in the matrix and counteract the possible expanding effects caused by the solvent. It can be seen in the Pareto diagram (Figure 4) that the expansion effect of the impregnation method (D) is generally significative and positive. Thus, PSI achieved greater permanent swelling effects than SSI. Although, the significance of the relationship between the type of extract and the impregnation method (CD) acquires a higher degree of significance. In the SEM images (Figure 5), a similar surface in the two samples impregnated with the hydroethanolic extract can be observed, so the impregnation method does not seem to be relevant in the structure of the polymer. However, with the ethanolic extract, a difference in the geometry of the pores can be observed. The generated pores in the SSI appear narrower and larger than in the PSI. Some cracks appeared in the SSI with ethanolic GPE, too.

In all the cases, a greater expansion was observed when increasing temperature at isobaric conditions. This could be explained by the lower density and viscosity of scCO_2_ at higher temperatures, which improves its diffusivity and therefore its sorption in the polymer [13]. Pressure had a lesser impact on the structure of the polymer, even if higher pressure levels under isothermal conditions would result in slightly greater expansion. In this case, when the pressure goes up, the density of scCO_2_ also increases and its diffusivity is reduced, but, at the same time, its solubility into PLA increases. These opposite effects mean that the swelling of the polymer will depend on the specific conditions used for the impregnation process.

**Figure 6:**
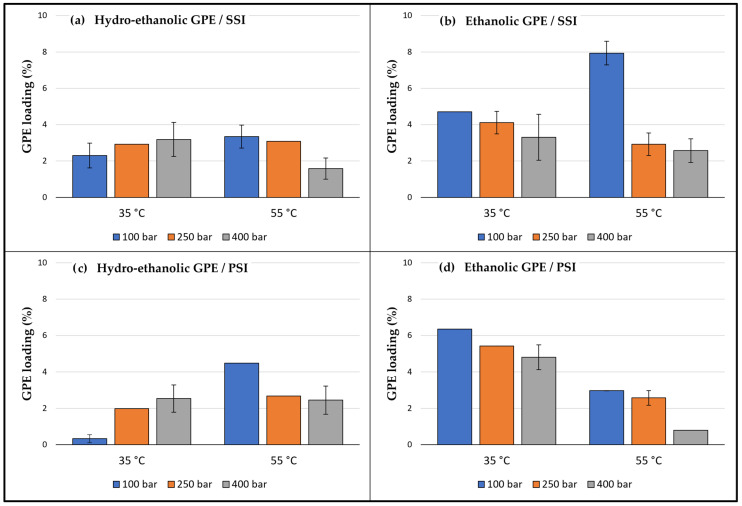
Grape pomace extract loadings in PLA depending on type of GPE (hydroethanolic or ethanolic), impregnation method (SSI or PSI), and temperature and pressure conditions. Results are represented by the mean of three values and standard deviation.

**Figure 7:**
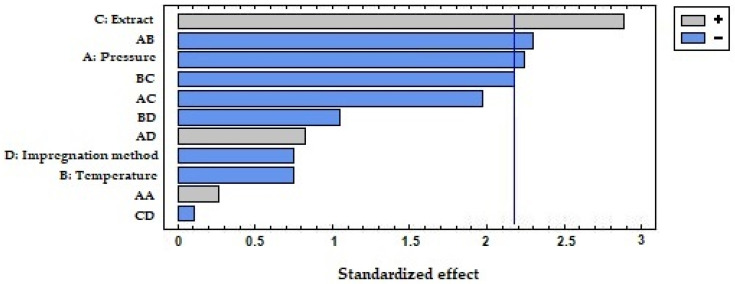
Standardized Pareto chart of the loading of grape extract pomace into polylactic acid samples. Effects: pressure (A), temperature (B), type of extract (C), impregnation method (D), and two-factor interactions.

After the expansion of the samples, the amount of extract loaded into the polymer was evaluated. Figure 6 displays the results, where it can be seen that, in the same way as for the expansion of the polymers, the type of extract was again the most influential variable for the loadings achieved (this can also be verified in the Pareto diagram in Figure 7). For most conditions, the amounts of ethanolic GPE impregnated were greater than those corresponding to the hydroethanolic GPE. This difference is dependent on the complex interactions that take place between the active compounds, the solvents, including scCO_2_, and the polymer. In the case of SSI, carbon dioxide is the element responsible for the solubilization of the active compounds, which had been initially diluted in ethanol and water, and for their transport into the polymeric matrix. So, the competition between the different solvents to dilute these active compounds plays a fundamental role in this process. Thus, although polar compounds like polyphenols are not very soluble in carbon dioxide, it has been demonstrated that the presence of organic solvents improves their solubility. For example, catechins, which are present in both extracts, are better solubilized by CO_2_ when ethanol is added as a cosolvent [50]. However, in the hydroethanolic extract, the polyphenol molecules could be found linked to the water molecules through hydrogen bonds, which hindered their solvation into the supercritical medium, and although the hydroethanolic extract had a greater concentration in polyphenols, the impregnation loads were lower than those corresponding to the ethanolic extract. Given that the most influential factors with regard to impregnation loads for the PSI method are the swelling of the polymer and the ability of the solvent to transport the active compounds into the polymer matrix, and that the ethanolic extract has previously demonstrated a greater swelling, this could explain why the ethanolic GPE achieved greater loading than the hydroethanolic one. Regarding chemical affinity, the hydroxyl groups of polyphenols may be linked by hydrogen bonds with the carbonyl groups of the PLA [15]. However, it should be noted that there is a competition against the intermolecular forces that hold these polyphenols linked to their starting solvents. This force can be considerably greater in the hydroethanolic extracts, which may explain why their impregnation loads were lower.

A combined effect between pressure, temperature, and type of extract has also been observed, and it follows similar trends in both of the two impregnation methods. Thus, at constant lower temperatures, hydroethanolic GPEs achieved greater loadings as the pressure was increased. However, at higher temperature levels, when the pressure was increased, the loadings were poorer. On the other hand, in the case of the ethanolic GPEs, increasing pressure while temperature remained invariable always meant a reduction in the loads obtained. Then, under isobaric conditions, at low pressure, greater loads of impregnated hydroethanolic GPE were obtained as the temperature was increased. Contrarily, lower loads were registered when temperature went up at higher pressure levels for the same extract. And for the ethanolic GPE, an increase in temperature, under isobaric conditions, always meant a decrease in loadings, except for the condition at 50 °C and 100 bar by SSI. In fact, among all the conditions tested, the impregnation of the ethanolic extract by SSI at 55 °C and 100 bar stands out for obtaining the highest load, around 8% of the impregnated extract. Rosales and coworkers [14] obtained the highest loading of ethanolic mango leaf extract into PLA by SSI under the same conditions (55 °C and 100 bar) with approximately 9% of the extract loaded. However, the same polymer impregnated with an ethanolic olive leaf extract by SSI under the same conditions only reached a loading of 0.5%, and for the best conditions (55 °C and 400 bar), the impregnation yield did not exceed 4% [15].

**Figure 8:**
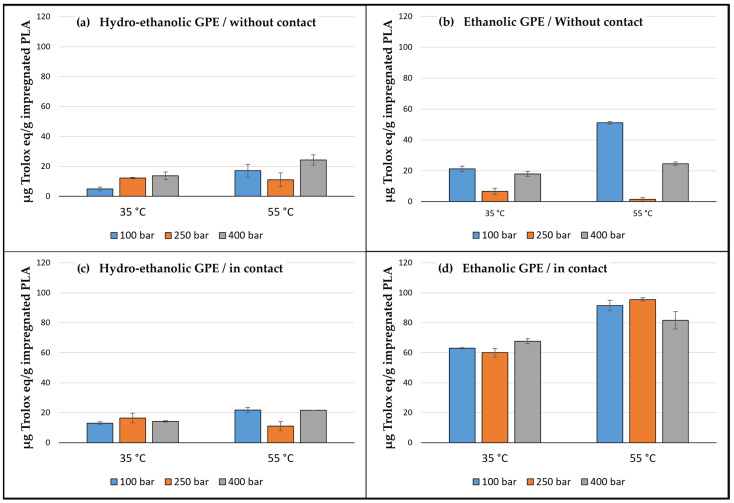
Antioxidant activity of the impregnated PLA samples depending on the type of GPE, the impregnation method, and the temperature and pressure conditions. Results are represented by the mean of three values and standard deviation.

**Figure 9:**
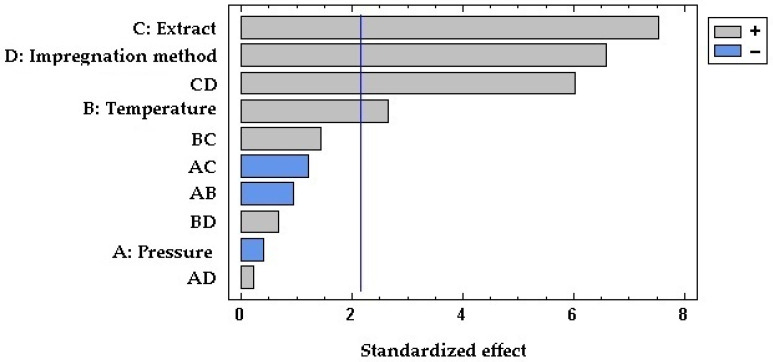
Standardized Pareto chart of the antioxidant capacity of the polylactic acid samples impregnated with grape pomace extract. Effects: pressure (A), temperature (B), type of extract (C), impregnation method (D), and two-factor interactions.

The bioactivity of the impregnated devices obtained was evaluated by studying the antioxidant activity through the DPPH method. The equivalent quantity of Trolox released by every sample during the assay is shown in Figure 8. The samples impregnated with the ethanolic GPE had a higher antioxidant capacity, especially when the polymer was in contact with the extract. Thus, these two qualitative variables, type of extract and impregnation method, had a substantial influence on the antioxidant activity of the devices, as can be seen in the Pareto chart in Figure 9. The greater the amount of extract impregnated, the greater the antioxidant activity of the devices, like in the case of the samples impregnated with ethanolic extract by PSI at low temperatures. It must be taken into account that, for this assay, the antioxidant activity was quantified by submerging the impregnated samples into a DPPH solution and then measuring the inhibition of the oxidation achieved by the extract released into the medium over the 90 min of the assay. Because the release rate depends to a large extent on the porosity reached by the polymer sample during its impregnation stage [13], the samples impregnated with ethanolic extract by PSI at the highest temperature, with a greater swelling and porosity, exhibited a higher antioxidant activity despite their lower impregnation loadings.

With respect to the operating conditions, an increase in temperature positively affected the swelling effect and, in turn, the antioxidant activity, in agreement with that explained above. In contrast, pressure had no significance either on the swelling effect or on the antioxidant activity.

## 4. Conclusions

This work delves into the prospective use of polylactic acid polymers functionalized by means of natural extracts for biomedical purposes. Specifically, two extracts from red grapes, obtained using CO_2_ and pressurized cosolvents, have been applied. The employment of a water–ethanol mixture as a cosolvent allowed for an extract with a higher concentration of polyphenolic compounds and a higher proportion of flavanols to be obtained, in comparison with the extract obtained when ethanol was used as the only solvent. Although both extracts presented excellent antioxidant, anti-inflammatory, and antimicrobial properties, making them good candidates for use in biomedical applications, the hydroethanolic extract was the most effective. However, after their impregnation into the PLA samples, the ethanolic extract obtained better results both in terms of loading and antioxidant activity, partly due to the greater swelling effect of the polymer achieved by this extract.

The results obtained by the supercritical CO_2_ (SSI) impregnation method were compared against those obtained through a new high-pressure soaking impregnation method (PSI) and, although the differences were not significant in terms of the loadings achieved, PSI stands out with regard to the antioxidant capacity of the resulting devices. Thus, this new method poses a rather promising foresight in the impregnation of medium–high polarity compounds.

## Figures and Tables

**Table 1:** Some chemical reagents and microbial strains used in this work.

Use	Reagent	Supplier
Extraction and impregnation	CO_2_ (99%) Partially denatured ethanol (97%)	Abelló Linde (Barcelona, Spain) Alcoholes del Sur (Córdoba, Spain)
Liquid chromatography	Acetonitrile (HPLC grade) Methanol (HPLC grade)	Panreac Química (Barcelona, Spain)
	(+)-Catechin, Quercetin, and Cyanidin chloride (analytical standards)	Sigma-Aldrich (Steinheim, Germany)
Antioxidant activity	2,2-diphenyl-1-picrylhydrazyl (DPPH) 2,2′-Azino-bis(3-ethylbenzothiazoline-6-sulfonic acid) diammonium salt (ABTS) Potassium persulfate (K_2_S_2_O_8_) (±)-6-Hydroxy-2,5,7,8-tetramethylchromane-2-carboxylic acid (Trolox)	Sigma-Aldrich (Steinheim, Germany)
Anti-inflammatory tests	Albumin from chicken egg (lyophilized powder) Lipoxidase from Glycine max (soybean) Linoleic acid Xylenol orange disodium salt	Sigma-Aldrich (Steinheim, Germany)
Antimicrobial activity	*Staphylococcus aureus* (ATCC 6538)	Microbiologics (Saint Cloud, MN, USA)
	Tryptic Soy Broth (TSB) 2,3,5,-triphenyltetrazoliumchloride (TTC)	Sigma-Aldrich (Steinheim, Germany)

**Table 2:** Calibration lines for target compounds analyzed by UPLC-ESI-ToF-MS.

Analyzed Compound (Group of Compounds)	Calibration Line
Catechin (Flavanols and proanthocyanidins)	$y=-33.329x^{2}+7716.5x\;\;\;(R^{2}=0.999)$
Quercetin (Flavonols)	$y=-153.17x^{2}+28,702x\;\;\;(R^{2}=0.995)$
Cyanidin (Anthocyanins)	y=343.61x (R=0.992)

**Table 3:** Design of experiments for the impregnation of GPEs into PLA.

Variable	Levels		
	Low	Medium	High
Pressure (A)	100 bar	250 bar	400 bar
Temperature (B)	35 °C	-	55 °C
Extract (C)	Hydroethanolic GPE	-	Ethanolic GPE
Impregnation method (D)	SSI	-	PSI

**Table 4:** Concentration of phenolic compounds (except anthocyanins) found in every grape pomace extract by UPLC-ESI-ToF-MS.

	RT	m[M-H]^−^	[E-GPE]	[HE-GPE]
Flavanols				
Procyanidin B1 (C_30_H_26_O_12_)	2.38	577.135	n.d.	7.6 ± 1.1
Procyanidin B2 (C_30_H_26_O_12_)	2.75	577.135	1.4 ± 0.2	6.6 ± 1.0
Procyanidin B3 (C_30_H_26_O_12_)	3.60	577.135	0.9 ± 0.1	3.8 ± 0.6
Procyanidin B4 (C_30_H_26_O_12_)	3.88	577.135	1.0 ± 0.1	3.9 ± 0.7
Catechin (C_15_H_14_O_6_)	3.06	289.072	11.0 ± 2.1	37.7 ± 7.5
Epicatechin (C_15_H_14_O_6_)	4.32	289.072	5.9 ± 1.1	33.4 ± 6.7
Flavonols				
Quercetin-3-*O*-galactoside (C_21_H_20_O_12_)	6.04	463.088	0.5 ± 0.1	1.9 ± 0.4
Quercetin-3-*O*-glucuronide (C_21_H_18_O_13_)	6.10	477.067	2.8 ± 0.4	2.5 ± 0.5
Quercetin-3-*O*-glucoside (C_2 1_H_20_O_12_)	6.18	463.088	3.0 ± 0.6	8.7 ± 1.7
Quercetin-3-*O*-rhamnoside (C_21_H_20_O_11_)	6.38	447.093	0.1 ± 0.0	0.3 ± 0.0
Kaempherol-3-*O*-galactoside (C_21_H_20_O_11_)	6.60	447.093	0.4 ± 0.1	0.6 ± 0.1
Kaempherol-3-*O*-glucoside (C_21_H_20_O_11_)	6.86	447.093	1.1 ± 0.2	2.1 ± 0.3
Isorhamnetin-3-*O*-galactoside (C_21_H_18_O_13_)	6.95	477.103	0.9 ± 0.1	0.5 ± 0.1
Isorhamnetin-3-*O*-glucoside (C_21_H_18_O_13_)	7.08	477.103	2.6 ± 0.5	5.0 ± 0.7

RT: retention time; m[M-H]^−^: monoisotopic mass of the negative ion; [E-GPE]: concentration of the compound in the ethanolic grape pomace extract (mg/L); [HE-GPE]: concentration of the compound in the hydroalcoholic grape pomace extract (mg/L); n.d. = not detected.

**Table 5:** Concentration of anthocyanins found in ethanolic and hydroethanolic grape pomace extracts by UPLC-ESI-ToF-MS.

	RT	m [M]^+^	[E-GPE]	[HE-GPE]
Malvidin 3-*O*-glucoside (C_23_H_25_O_12_^+^)	5.77	493.135	148.9 ± 22.3	587.8 ± 88.2
Delphinidin-3-*O*-glucoside (C_21_H_21_O_12_^+^)	6.68	465.103	91.0 ± 13.7	265.5 ± 39.8
Malvidin-3-O-(6-*O*-acetyl)glucoside (C_25_H_27_O_13_^+^)	6.81	535.145	188.2 ± 28.2	558.1 ± 83.7
Malvidin-3-O-(6-*O*-caffeoyl)glucoside (C_32_H_31_O_15_^+^)	7.05	655.166	43.6 ± 6.5	105.3 ± 15.8
Cyanidin-3-*O*-glucoside (C_21_H_21_O_11_^+^)	7.12	449.108	36.0 ± 5.4	75.7 ± 11.3
Petunidin-3-*O*-glucoside (C_22_H_23_O_12_^+^)	7.22	479.119	54.1 ± 8.1	132.7 ± 19.9
Malvidin-3-(6-*O*-coumaroyl)glucoside (C_32_H_31_O_14_^+^)	7.51	639.171	177.1 ± 26.6	401.2 ± 60.2

RT: retention time; m[M]^+^: monoisotopic mass of the positive ion; [E-GPE]: concentration of the compound in the ethanolic grape pomace extract (mg/L); [HE-GPE]: concentration of the compound in the hydroalcoholic grape pomace extract (mg/L)

**Table 6:** Bioactivity of grape pomace extracts.

	Ethanolic GPE	Hydroethanolic GPE
Antioxidant capacity		
DPPH Assay (mg TE/g dry extract)	0.24 ± 0.03	0.50 ± 0.03
IC50 (µg/mL)	168.2 ± 14.2	77.6 ± 4.0
ABTS Assay (mg TE/g dry extract)	0.31 ± 0.01	0.66 ± 0.06
IC50 (µg/mL)	100.9 ± 1.8	46.3 ± 1.0
Anti-inflammatory capacity		
Protein denaturation inhibition IC50 (µg/mL)	2126 ± 201	1015 ± 65
Lipoxygenase activity inhibition IC50 (µg/mL)	473 ± 35	1350 ± 20
Antibacterial capacity		
*S. aureus* MIC (IC90) (µg/mL)	17.0 ± 1.7	18.2 ± 0.7

## Data Availability

Not applicable.
